# International Society of Sports Nutrition Position Stand: Long-Chain Omega-3 Polyunsaturated Fatty Acids

**DOI:** 10.1080/15502783.2024.2441775

**Published:** 2025-01-15

**Authors:** Ralf Jäger, Jeffery L. Heileson, Sidney Abou Sawan, Broderick L. Dickerson, Megan Leonard, Richard B. Kreider, Chad M. Kerksick, Stephen M. Cornish, Darren G. Candow, Dean M. Cordingley, Scott C. Forbes, Grant M. Tinsley, Tindaro Bongiovanni, Roberto Cannataro, Bill I. Campbell, Shawn M. Arent, Jeffrey R. Stout, Douglas S. Kalman, Jose Antonio

**Affiliations:** a Increnovo LLC, Whitefish Bay, WI, USA; b Walter Reed National Military Medical Center, Nutrition Services Division, Bethesda, MD, USA; c Department of Health, Human Performance, and Recreation, Baylor University, Waco, TX, USA; d Iovate Health Sciences International, Oakville, Canada; e Exercise & Sport Nutrition Lab, Human Clinical Research Facility, Department of Kinesiology and Sport Management, Texas A&M University, College Station, TX, USA; f Exercise and Performance Nutrition Laboratory, College of Science, Technology, and Health, Lindenwood University, St. Charles, MO, USA; g Faculty of Kinesiology and Recreation Management, University of Manitoba, Winnipeg, Canada; h Faculty of Kinesiology and Health Studies, University of Regina, Regina, Canada; i Applied Health Sciences Program, Faculty of Graduate Studies, University of Manitoba, Winnipeg, Canada; j Department of Physical Education Studies, Brandon University, Brandon, Canada; k Department of Kinesiology and Sport Management, Texas Tech University, Lubbock, TX, USA; l Department of Biomedical and Neuromotor Sciences (DIBINEM), University of Bologna, Bologna, Italy; m Player Health & Performance Department, Palermo Football Club, Palermo, Italy; n GalaScreen Laboratories, Department of Pharmacy, Health and Nutritional Sciences, University of Calabria, Rende, Italy; o Research Division, Dynamical Business & Science Society – DBSS International SAS, Bogotá, Colombia, USA; p Performance& Physique Enhancement Laboratory, Exercise Science Program, University of South Florida, Tampa, FL, USA; q Department of Exercise Science, Arnold School of Public Health, University of South Carolina, Columbia, SC, USA; r School of Kinesiology and Rehabilitation Sciences, University of Central Florida, Orlando, FL, USA; s Dr. Kiran C. Patel College of Osteopathic Medicine, Nova Southeastern University, Davie, FL, USA; t Department of Health and Human Performance, Nova Southeastern University, Davie, FL, USA

**Keywords:** Omega-3, fatty acids, performance, exercise, sleep, inflammation

## Abstract

Position Statement: The International Society of Sports Nutrition (ISSN) presents this position based on a critical examination of the literature surrounding the effects of long-chain omega-3 polyunsaturated fatty acid (ω-3 PUFA) supplementation on exercise performance, recovery, and brain health. This position stand is intended to provide a scientific foundation for athletes, dietitians, trainers, and other practitioners regarding the effects of supplemental ω-3 PUFA in healthy and athletic populations. The following conclusions represent the official position of the ISSN:

1. Athletes may be at a higher risk for ω-3 PUFA insufficiency.
2. Diets rich in ω-3 PUFA, including supplements, are effective strategies for increasing ω-3 PUFA levels.
3. ω-3 PUFA supplementation, particularly eicosapentaenoic acid (EPA) and docosahexaenoic acid (DHA), has been shown to enhance endurance capacity and cardiovascular function during aerobic-type exercise.
4. ω-3 PUFA supplementation may not confer a muscle hypertrophic benefit in young adults.
5. ω-3 PUFA supplementation in combination with resistance training may improve strength in a dose- and duration-dependent manner.
6. ω-3 PUFA supplementation may decrease subjective measures of muscle soreness following intense exercise.
7. ω-3 PUFA supplementation can positively affect various immune cell responses in athletic populations.
8. Prophylactic ω-3 PUFA supplementation may offer neuroprotective benefits in athletes exposed to repeated head impacts.
9. ω-3 PUFA supplementation is associated with improved sleep quality.
10. ω-3 PUFA are classified as prebiotics; however, studies on the gut microbiome and gut health in athletes are currently lacking.

## 1. Methods

ISSN position stands are invited papers the ISSN editors and Research Council identify as topics of interest to our readers that need position stands to provide guidance to readers and the profession. Editors and/or the Research Council identify a lead author or team of authors to perform a comprehensive literature review. The draft is then sent to leading scholars for review and comment. The paper is then revised as a consensus statement and reviewed and approved by the Research Council and Editors as the official position of the ISSN.

## 2. Introduction

Like all fatty acids, polyunsaturated fatty acids (PUFAs) consist of long chains of carbon atoms, with a carboxyl group at one end and a methyl group at the opposite end. PUFAs are characterized by having two or more double bonds between carbon atoms in the fatty acid chain, which distinguishes them from saturated and monounsaturated fatty acids. The two main classes of PUFAs are omega-3 (ω-3) and omega-6 (ω-6) fatty acids. ω-3 PUFAs are named for the first double bond, which appears on the third carbon from the methyl group (the omega end) of the fatty acid chain. Notable short-chain ω-3 PUFAs are alpha-linolenic acid (ALA; 18:3n-3) and stearidonic acid (18:4n-3), both of which contain 18 carbon atoms and feature three or four carbon-to-carbon double bonds. Long-chain ω-3 PUFAs, which contain more than 19 carbon atoms, include eicosapentaenoic acid (EPA; 20:5n-3), docosapentaenoic acid (DPA; 22:5n-3), and docosahexaenoic acid (DHA; 22:6n-3) [1,2].

ω-3 PUFAs exhibit functionally important cellular roles as they are part of the phospholipid bilayer of cellular membranes and are precursors to bioactive signaling molecules. DHA is present in high concentrations in the brain, retina, and sperm cells, indicating not only the bioenergetic roles of ω-3 PUFAs but functional roles as well [3–5]. ω-3 PUFAs are anti-inflammatory, anti-arrhythmic, and anti-thrombotic compared to ω-6 PUFAs, which demonstrate proinflammatory and prothrombotic properties. DHA and EPA serve as precursors for the production of mediators that downregulate inflammation, specifically resolvins, maresins, and protectins. These mediators modulate key controllers of inflammation, such as Nuclear Factor-κB (NF-kB), which, when activated, increases inflammation, and regulate the expression of Nuclear Respiratory Factor 1 (Nrf1), which plays a role in cellular defenses against oxidative [6].Consuming ω-3 PUFAs can exert protective effects on the cardiovascular, retinal, musculoskeletal, and cerebrovascular systems, and positively affect neurological disorders and conditions [7–9].

**Figure 1.**
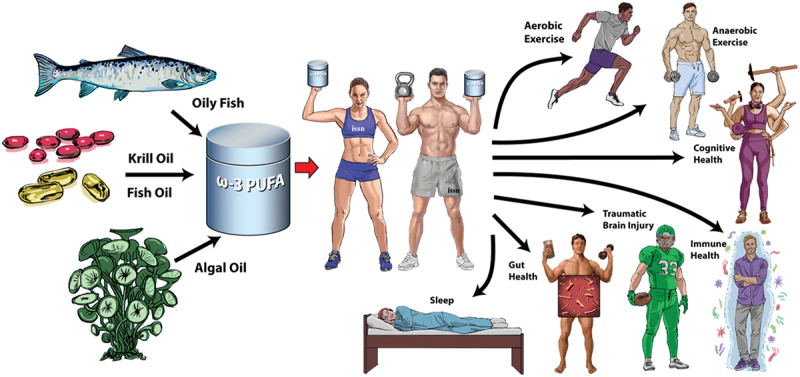
Potential health benefits of ω-3 PUFA supplementation in athletes (illustration by Stephen Somers, Milwaukee, WI, USA).

Purported sport-specific benefits of ω-3 PUFA supplementation may include reduced oxygen cost (e.g. improved exercise economy), immune system support, enhanced recovery, and improved anabolic responses to amino acids with and without training, especially in older adults and strength/power athletes. Furthermore, ω-3 PUFAs may positively influence digestive health, cognitive function, and sleep quality and provide protective effects against traumatic brain injuries (TBI) in athletes (Figure 1).

Athletes are typically at risk of ω-3 PUFA inadequacy. For example, National Collegiate Athletic Association Division I football athletes have suboptimal DHA and EPA levels. A 2019 study of 404 collegiate football players did not find a single athlete with an ω-3-index (O3i), a measurement of DHA and EPA content in erythrocytes expressed as a percentage of total fatty acids, greater than 8%, which is the value associated with the lowest risk for cardiovascular disease. Moreover, the average O3i for all 404 participants was 4.4 ± 0.8% indicating that these football players could be at a higher risk for cardiovascular disease later in life [10]. To raise the O3i from the observed values to the target value of 8%, an additional daily intake of about 1.4 g of EPA and DHA is needed, either through increased consumption of oily fish or ω-3 PUFA supplements [11].

## 3. Sources of ω-3 PUFA

Humans and other mammals can synthesize saturated fatty acids and some monounsaturated fatty acids from carbohydrates and protein-derived carbon groups. However, they lack the necessary desaturase enzymes to introduce a cis double bond at the *n*-6 or *n*-3 positions of fatty acids. As a result, ω-3 and ω-6 polyunsaturated fatty acids (PUFAs) are essential nutrients that must be obtained through the diet. Humans can synthesize ω-6 PUFA, such as arachidonic acid (AA; 20:4n-6), from linoleic acid (LA; 18:2n-6), and ω-3 PUFA, including EPA and DHA, from ALA. Research on ALA metabolism in healthy young men showed that approximately 5–10% of dietary ALA was converted to EPA, while conversion to DHA ranged from 2 to 5% [12]. Due to the low rate of ALA conversion into EPA and DHA, these ω-3 PUFAs are considered conditionally essential nutrients. Additionally, genetic variability and sex differences in enzyme activity affect an individual’s capacity to produce ω-3 PUFAs [13].

The most effective way to consume sufficient amounts of ω-3 PUFAs is by eating oily fish, such as salmon, mackerel, trout, sardines, and sea bass, which are the primary dietary sources of EPA and DHA, along with ω-3 PUFA fortified foods and ω-3 PUFA supplements. Plant-based foods rich in ALA include flaxseeds and flaxseed oil, chia seeds, and walnuts [14]. Dietary supplements can contain various forms of ω-3 PUFAs, including natural triglycerides, free fatty acids, ethyl esters, re-esterified triglycerides, and phospholipids. Natural triglycerides are the form of ω-3 PUFAs found naturally in fish oil, whereas krill oil primarily contains ω-3 PUFAs in the form of phospholipids. Ethyl esters are created by replacing the glycerol molecule in natural triglycerides with ethanol, while re-esterified triglycerides are produced by converting ethyl esters back into triglycerides. Although esterified triglycerides, natural triglycerides, and free fatty acids have slightly higher bioavailability than ethyl esters, phospholipids exhibit greater bioavailability than triglycerides. Nevertheless, consuming all forms of ω-3 PUFAs effectively increases plasma EPA and DHA levels [13]. Fish oil, krill oil, and algal oil are the main sources of ω-3 PUFAs in dietary supplements.

### 3.1. Fish oil

Aside from consuming oily fish, which predominates in countries like Japan and regions of Scandinavia, fish oil supplementation is the most popular method of obtaining EPA and DHA [15]. Common sources of commercial fish oils include salmon oil, mackerel oil, anchovy oil, and cod liver oil, all of which contain high concentrations of ω-3 PUFAs. Standard fish oils typically contain 180 mg of EPA and 120 mg of DHA per 1,000 mg, resulting in a total ω-3 PUFA content of about 30%. Fish oil supplements have been supported by extensive evidence from randomized controlled trials (RCTs), as highlighted in reviews and meta-analyses, demonstrating cardioprotective, antithrombotic, anti-inflammatory, and neuroprotective benefits [16,17].

### 3.2. Krill oil

Krill oil is another widely used form of ω-3 PUFA supplement. It is extracted from Antarctic krill, a microscopic shrimp that is part of the zooplankton and forms the base of the marine food chain, making it a rich source of ω-3 PUFAs [18]. Krill oil is similar to fish oil in that it contains DHA and EPA. In contrast to fish oil, krill oil contains most of the fatty acids in phospholipid form (most notably phosphatidylcholine) [19]. It is theorized that ω-3 PUFAs in phospholipid form can aid the passage of fatty acids through the intestinal wall and bolster the bioavailability of these fatty acids since phospholipids make up the structure of the cell membrane [19]. Krill oil also contains the antioxidant astaxanthin, which could confer additional benefits [20]. Additionally, krill oil is generally considered to be less contaminated by dioxins and heavy metals compared to fish oil as krill are at the base of the food chain having a shorter lifespan and consume smaller organisms, which reduces the accumulation of toxins like dioxins and heavy metals. However, it’s important to note that levels of toxins in both krill oil and fish oil are typically very low due to purification processes during extraction.

### 3.3. Algal oil

Algae represent a diverse range of photosynthetic unicellular and multicellular organisms. Although microalgae are among the oldest life forms on Earth, recent research has focused on their consumption and its effects on human health [21,22]. Algal oil, which is rich in DHA and EPA, can be extracted from algal biomass grown in controlled fermentation vessels. Depending on the algae strain, algal oil often contains more DHA than EPA, while fish oil generally contains both DHA and EPA in a relatively balanced amount. Compared to fish oil, algal oil is more sustainable, has a lower risk of contaminants typically found in ocean waters, and is entirely suitable for vegetarian diets [23,24].

### 3.4. Key findings ω-3 PUFA

- ω-3 PUFA (i.e. EPA and DHA) are conditionally essential nutrients.
- Fatty fish and dietary supplements are both effective in raising ω-3 PUFA levels.
- Athletes belong to the groups at higher risk of ω-3 PUFA inadequacy.

## 4. Consensus and findings

### 4.1. Aerobic exercise

Endurance-type exercise typically involves low to moderate intensity and prolonged duration activities that rely heavily on aerobic metabolism and require sustained endurance capacity. On the other hand, resistance exercise is characterized by high-intensity and short-duration activities that rely primarily on anaerobic metabolism and require high levels of strength and power. While both types of exercise have been shown to be beneficial for overall health and fitness [25], the nutritional requirements and supplementation strategies for optimal performance can differ [26,27]. Specifically, ω-3 PUFAs have been shown to differentially impact resistance and endurance exercise performance with evidence suggesting that ω-3 PUFAs may enhance endurance capacity [28,29], primarily through sarcolemma [30], mitochondrial [31] and cardiovascular remodeling [32]. Early studies highlight potential physiological benefits of ω-3 PUFAs, such as enhanced red blood cell deformability (RCD) in hypoxic conditions. For example, Guezennec et al. [33] demonstrated that a fish oil-rich diet could prevent the typical reduction in RCD during exertion at altitude, suggesting improved blood flow in hypoxic environments, which may benefit athletes training or competing at high altitudes. Similarly, Oostenbrug et al. [34] examined the combination of fish oil and vitamin E on RBC characteristics in cyclists, noting some reduction in exercise-induced oxidative stress, despite no impact on RCD or performance. Nonetheless, here we discuss the role of ω-3 PUFAs from a muscle-centric view and how this can translate into whole-body endurance-exercise adaptations and performance. Key studies are summarized in Table 1.

**Table 1.** Summary of studies investigating the effect of ω-3 PUFA supplementation on endurance exercise.

Author (Year)	Athletes	Protocol/Season	Diet Control	EPA/DHA (mg/d) Dose and Duration	Timing	Outcome
Leaf and Rauch (1988)	12 trained athletes	Predicted VO_2max_	No	High–4200/1800 Low–2100/900 42 days	Daily	RBC deformability not reported ↑estimated VO_2max_ in the low group only
Guezennec et al. [33]	14 recreational trained males	1 h cycling at 70% of VO_2max_ at sea level and at a simulated altitude of 3000 m in a hypobaric chamber	No	1080/720 42 days	Daily	Attenuated RBC deformability loss during hypoxia ↑VO_2max_ during hypoxia
Brilla et al. (1990)	32 sedentary men	Exercise groups performed aerobic exercise for one hour three x per week.	No	4000* 70 days	Daily	↔ VO_2max_, ↔ VAT
Raastad et al. [44]	28 trained soccer players	VO_2max_, anaerobic threshold and running performance until exhaustion	No	1600/1040 70 days	Daily	↔ RBC osmotic fragility ↔ VO2max, max aerobic power ↔ Running performance
Oostenbrug et al. [34]	24 trained athletes	Trial performance, RBC characteristics, and lipid peroxidation	No	1020/720 21 days	Daily	↔ RBC deformability ↔ VO2max ↔ 70% VO2max cycling time trial
Huffman et al. (2004)	10 recreationally trained men and women	VO2 max and jogging for 75 min at 60% ṽO_2max_	No	2400/0 28 days	Daily	↔ HR ↔ VO_2_ uptake ↔ RPE ↔ TTF
Peoples et al. [45]	16 well-trained cyclists	Off season training VO_2peak_ and cycling at 55% of the peak workload until exhaustion	No	800/2400 56 days	Daily	↓ HR, VO_2_, RPP during cycling 55% VO_2max_ Performance ↔ VO2max ↔ Time to fatigue 55% VO2max)
Bloomer et al. (2009)	14 recreationally trained men	60 min treadmill climb using a weighted pack	Diet logs recorded	2224/2208 42 days	Twice per day (morning and evening) with meals.	↔ HR, ↔ VO2 uptake ↔ RPE
Buckley et al. [52]	25 well-trained football players	Pre-season Performance time for a 2200 m running time-trial	Habitual diet under supervision of the club dietitian.	360/1560 35 days	Daily	↓ HR during 10 km treadmill run ↔ TT (2.2 km run)
Nieman et al. [142]	16 cyclists	3-d period in which they cycled for 3 hr/d at ~57% Wmax with 10-km time trials inserted during the final 15 min of each 3-hr bout	Dietitian instructed the participants to follow a diet moderate in carbohydrate the weekend before and during the 3-day exercise period.	2,000/400 42 days	Twice per day, (morning while fasted and evening before a meal).	↔ TT (10 km)
Boss et al. [46]	16 sedentary men	VO_2max_ and time trial at 80 % VO_2max_.	Yes	1100/700 28 days	3 times per day with each meal.	↔ VO_2max_, ↔ TTE, ↔ max aerobic power
Macartney et al. [50]	28 trained athletes	4 part protocol 1. 10 min submaximal cycling at 125 W 2. 6 × 30 s Wingate cycling sprints/150 s recovery 3. 5 min work capacity trial 4. supine recovery	No	140/540 56 days	Daily	↓ submaximal HR, faster HR recovery ↔ 6 × 30 s power output ↔ 5 min cycling time trial
Kawabata et al. [53]	20 recreationally trained men	VO_2max_ and submaximal exercise at 30 minutes at 2 and 3 mm BLa with 10 minutes rest between submaximal exercise	No	914/399 56 days	3 times per day with each meal.	↑ VO_2_ during submaximal exercise
Zebrowska et al. [29]	13 well-trained cyclists	Pre-season	No	660/440 21 days	Twice daily with meals	↑ FMD, ↑ NO release ↑ VO_2max_, ↑max aerobic power
Da Boit et al. [137]	37 men and women	Participants cycled at self-selected cadence (70–90 rpm) with workload increasing by 30 Watts every minute for males and 20 Watts every minute for females until volitional exhaustion.	No	240/120 42 days	Daily	↔ HR, ↔ VO_2_ ↔ TT (cycling)
Haghravan et al. [82]	44 women	VO2max	Diet education and 24-hour food records at baseline week 4 and 8	600/300 56 days	Daily	↑ VO_2max_
Avila-Gandia et al. (2020)	38 amateur cyclists	Graded cycling until exhaustion	7 days diet log during the first week	120/975 30 days	Daily before breakfast	↔ HR, ↑ HR recovery, ↑ VO_2_ VO_2max_, ↑ power output
Serajian et al. (2021)	18 recreationally, active men	Neuromuscular performance after time trial to exhaustion	FFQ	1320/660 28 days	3 times per day with each meal.	↔ M-wave ↓ RPE ↔ MVC after exhaustive exercise
Tomczyk et al. [28]	26 amateur, male long-distance runners	Graded exercise test to exhaustion with assessment of ṼO_2peak_, running economy, and a 1500-m run trial	3 day diet logs recorded during the first and the last week of the program	2234/916 84 days	Twice per day (morning and evening)	↑ VO_2_ ↔ TT ↔ VO_2peak_

FMD = flow-mediated dilation; FFQ = food frequency questionnaire; HR = heart rate; MVC = maximal voluntary contraction; NO = nitric oxide; RBC = red blood count; RPE = rate of perceived exertion; TT = time trial; RPP = rate pressure product; TTF = time to fatigue; VAT = ventilatory anaerobic threshold; VO_2_max = maximal oxygen uptake; ↑ = significant increase; ↓ = significant decrease; ↔ = no significant difference.

Fish oil supplementation results in the incorporation of ω-3 PUFAs into skeletal and myocardial muscle membranes [35,36]. For example, four weeks of fish oil supplementation (3,500 mg EPA and 900 mg DHA) increases both blood and skeletal muscle ω-3 PUFA content [36]. Specifically, fish oil supplementation results in changes in muscle ω −3 PUFA composition of skeletal muscle within two weeks [36], compared to months for adipose tissue [35]. Furthermore, a 12-week supplementation regimen of 3,000 mg EPA and 2,000 mg DHA per day increases the total phospholipid content – major constituents of cell membranes – in both whole muscle and sarcolemma, but not in the mitochondria. However, sarcolemma membranes appear to be less responsive than whole muscle and mitochondria, likely due to the low ω-6/ω-3 PUFA ratio [30]. Importantly, the remodeling of the sarcolemma in response to ω-3 PUFA supplementation coincides with the cell membrane being the site to remodel muscle proteins after endurance exercise [37]. Furthermore, satellite cells, which play an important role in muscle regeneration after exercise, reside above the sarcolemma [38] and are activated in response to endurance exercise [39]. Given that ω-3 PUFAs are incorporated within the phospholipid cell membrane, a recent report also hypothesized that ω-3 PUFA supplementation can aid in muscle regeneration after exercise [40]. Finally, disuse studies reveal that incorporation of ω-3 PUFAs (3,000 mg EPA and 2,000 mg DHA) into mitochondrial membranes alters indices of mitochondrial bioenergetics, such as preserved adenosine diphosphate (ADP) sensitivity [31], which may subsequently impact energy metabolism during reloading. Collectively, these data suggest that ω-3 PUFA supplementation for a minimum of two weeks can remodel skeletal muscle phospholipids which may subsequently impact endurance capacity and performance.

Exercise economy, maximal oxygen uptake (VO_2_max), and lactate threshold are all strongly related to endurance exercise performance [41]. Furthermore, mitochondria are the cellular organelles responsible for energy production through oxidative phosphorylation, and their number and function are critical for sustaining aerobic metabolism during prolonged exercise [42,43]. Specifically, the ability of skeletal muscle to consume oxygen during exercise is a key determinant of endurance capacity [41]. To this end, a recent report revealed that 12 weeks of ω-3 PUFA supplementation (2,234 mg of EPA and 916 mg of DHA/day) during endurance training improves the O3i, running economy, and increases VO_2_peak in amateur runners [28]. These findings are in contrast to Raastad et al. [44] who showed no changes in VO_2_max and running performance in trained soccer players receiving 1,600 mg of EPA and 1,040 mg of DHA per day through a 10-week period. These results suggest that a higher dose of ω-3 PUFAs may be required to remodel the phospholipid to induce ergogenic effects. However, others have demonstrated that 3,200 mg of ω-3 PUFA (800 mg of EPA and 2,400 mg of DHA) supplementation per day over eight weeks increased ω-3 PUFA content of erythrocyte cell membranes, lowered heart rate during incremental workloads to exhaustion, and reduced whole-body and myocardial O_2_ demand during submaximal exercise (55% VO_2_peak) in well-trained men [45]. Boss et al. [46] found that a 10-day diet rich in fish and olive oils enhanced time to exhaustion performance (at 80 % VO_2_max) but not VO_2_max and improved insulin sensitivity with a trend to improve fat oxidation in healthy young untrained men. Thus, these data suggest that ω-3 PUFA supplementation is associated with improved exercise economy and aerobic capacity.

Cardiovascular function plays a crucial role in endurance exercise as it determines the delivery of oxygen and nutrients to working muscles and removal of metabolic waste products which enhances health [47] and performance [48]. A bout of endurance exercise induces a response in cardiovascular function, including an increase in heart rate, stroke volume, and cardiac output, as well as vasodilation of blood vessels – adaptations essential for maintaining a steady supply of oxygen and nutrients to working muscles during prolonged exercise [49]. Several studies have examined the effects of fish oil and ω-3 PUFA supplementation on cardiovascular function and exercise performance. For example, low-dose fish oil supplementation (140 mg EPA and 560 mg DHA) over eight weeks increased the ω-3 PUFA index, reduced mean exercise heart rate, and improved heart rate recovery without compromising peak heart rate in trained men [50]. A follow-up study examining a similar dose of ω-3 PUFA supplementation (containing 140 mg EPA and 560 mg DHA) on repeated bouts of physiologically stressful cycling and a subsequent time trial in a state of fatigue found no evidence of endurance performance enhancement in trained males, despite elevating the ω-3 PUFA index after eight weeks [51]. However, twice daily supplementation with ω-3 PUFA consisting of 660 mg EPA, 440 mg DHA and 200 mg of other acids can positively impact endothelial function and exercise performance [29]. Specifically, ω-3 PUFA supplementation increased baseline nitric oxide levels (NO) and flow-mediated dilation compared to placebo [29]. Additionally, there was a positive correlation between baseline post-intervention NO concentration and maximal oxygen uptake, and between ΔNO and ΔVO_2_max [29]. This increase in NO release in response to ω-3 PUFA supplementation may play a central role in cardiovascular adaptive mechanisms and enhanced exercise performance in cyclists, findings that also have been replicated in overweight participants [52]. Macartney et al. [50] showed that low-dose fish oil supplementation (containing 140 mg EPA and 560 mg DHA) for eight weeks improved heart rate recovery, indicating enhanced cardiac function in trained males. Furthermore, Kawabata et al. [53] reported improved exercise economy and reduced perceived exertion in response to eight weeks of EPA-rich fish oil (containing 914 mg EPA and 399 mg DHA) in recreationally active men, possibly through improved oxygen delivery. Collectively, these findings suggest that athletes may benefit from incorporating ω-3 PUFA supplementation into their diets to improve cardiovascular health; however, its direct impact on endurance performance remains inconsistent.

#### 4.1.1. Key findings for aerobic exercise

•Studies have shown that ω-3 PUFA supplementation can improve running economy, aerobic capacity, and cardiovascular function during endurance exercise.

- ω-3 PUFA can improve cardiovascular dynamics during and after exercise as evidenced by enhanced red blood cell deformability, endothelial function, and heart rate recovery; however, their direct impact on endurance performance remains inconsistent.
- The incorporation of ω-3 PUFA into skeletal muscle membranes has been found to result in changes in muscle ω-3 PUFA composition, particularly in the sarcolemma, which is essential for muscle remodeling and/or regeneration after endurance exercise.

### 4.2. Body composition, strength and power

Recently, ω-3 PUFAs have been linked to various aspects of physical performance and recovery [54–56]. The influence of ω-3 PUFAs on various physiological processes appears to be mediated by their incorporation into tissue phospholipid membranes [57]. While the exact mechanisms remain elusive, musculoskeletal benefits may stem from a reduction in pro-inflammatory cytokines, enhanced neural activation, reduced activation of pathways involved in protein degradation, improvement of insulin sensitivity, and reduction of mitochondrial reactive oxygen species emission [57,58].

Growing evidence from cell systems [59,60], pre-clinical animal models [61–65], and humans [24,66–70] demonstrate that ω-3 PUFAs modulate muscle protein metabolism and may influence skeletal muscle outcomes such as fat-free mass, strength, and power, especially in response to nutrient (e.g. protein) and mechanical stimuli (i.e. resistance training). In seminal human studies, Smith et al. [68,69] showed that ω-3 PUFA ingestion (4 g/d, containing 3,360 mg EPA+DHA) for eight weeks altered skeletal muscle fatty acid composition and increased rates of mechanistic target of rapamycin (mTOR) signaling and muscle protein synthesis (MPS) during periods of hyperaminoacidemia in healthy, younger [69] and older adults [68]. Follow-up ω-3 PUFA supplementation trials have reported potent treatment effects on strength [70,71] and fat-free mass [70,72]. In fact, the effect of ω-3 PUFA supplementation on skeletal muscle outcomes in older adults has been thoroughly explored [70,71,73–77]. Recent meta-analyses have reported positive effects on muscle function or strength [73,75,76,78], with or without resistance exercise training (RET), and one reported a significant increase in fat-free mass [73].

Despite strong mechanistic underpinnings and preliminary data in older adults, skeletal muscle outcome data in young adults remains less appreciated. This is partially due to the conflicting mechanistic evidence regarding anabolic signaling from high dose ω-3 PUFA supplementation in young adults. McGlory and colleagues [36] reported an increase in the proportion of ω-3 PUFA, especially EPA, in the muscle cell following four weeks of high-dose fish oil (5 g/d, containing 3,500 mg EPA and 900 mg DHA), which subsequently led to an increased phosphorylation of mTORC1 and focal adhesion kinase (nutrient- and mechanically-sensitive anabolic signals, respectively). However, a more recent investigation in resistance-trained young adults demonstrated that eight weeks of high-dose fish oil (5 g/d, containing 3,500 mg EPA and 900 mg DHA]) did not augment the MPS response to ingestion of 30 g of whey protein under both rested and post-exercise conditions [79]. Nonetheless, there appeared to be a trend for MPS to be greater in the fed state at rest (*d* = 0.77) and after exercise (*d* = 0.83) in the high-dose fish oil group, so it is unclear whether differences would have been found if the sample size was greater or if training status was similar between groups based on the differential baseline strength measures. While follow-up studies are needed to explore these complex relationships, a few studies have investigated the effect of ω-3 PUFA supplementation on fat-free mass, strength, and power in young adults (Table 2). Herein, we review the role of ω-3 PUFA on skeletal muscle adaptations in young adults.

**Table 2.** Summary of studies investigating the effect of ω-3 PUFA supplementation on body composition, strength and power.

First Author (year)	Participants	Exercise Protocol	Dose & Duration	LC ω-3 PUFA Tissue Measure	Body Composition	Strength/Power	Notes
***Resistance Training***							
Heileson (98)	Adult men and women Total: *n* = 21 PL: *n*=11 ω-3: *n*=10	Progressive RET, 3 full-body sessions∙wk^−1^	Fish oil Total dose: 4.5 g∙d^−1^ EPA: 2.28 g DHA: 1.58 g Duration: 10-wk	Yes, whole blood O3I: ↑ EPA: ↑ DHA: ↑	DXA FFM: ↔ FM: ↔	BP_1RM_ Absolute: ↑ Relative: ↑ BSQ_1RM_ Absolute: ↔ Relative: ↑	Single-blind RET only partially supervised Fishy burps reported (*n*=2)
Georges (81)	Adult men Total: *n* = 18 PL: *n*=9 ω-3: *n*=9	4 sessions∙wk^−1^	Krill oil Total dose: 0.96 g∙d^−1^ EPA: 0.39 g DHA: 0.24 g Duration: 8-wk	Unmeasured	DXA FFM: ↔ FM: ↔	BP_1RM_ ↔ LP_1RM_ ↔	Used krill oil Low dose Underpowered
Hayward (80)	Adult women Total: *n* = 28 PL: *n*=10 ω-3: *n*=8	Pre-training: 3 sessions∙wk^−1^ Training during intervention: 4 sessions∙wk^−1^	Fish oil Total dose: 0.9 g∙d^−1^ EPA: 0.54g DHA: 0.36g Duration: 4-wk	Unmeasured	DXA FFM: ↔ FM: ↔	BP_1RM_ ↔ BSQ_1RM_ ↔ DL_1RM_ ↔ HTh_1RM_ ↔	Short supplementation period Low dose Multi-variable intervention Open label
***Aerobic Training***							
Haghravan (82)	Adult women Total: *n*=44 PL: *n*=22 ω-3: *n*=22	Aerobic exercise	Fish oil Total dose: unreported EPA: 0.60 g∙d^−1^ DHA: 0.30 g∙d^−1^ Duration: 8-wk	Unmeasured	BIA FFM ↔ FM ↓	No measure	Minimal information provided regarding the type/duration of aerobic training
***No Standardized Exercise***							
Heileson (103)	Male and female athletes Total: *n*=27 PL: *n*=12 ω-3: *n*=15	In-season training	Fish oil Total dose: 3.0 g∙d^−1^ EPA: 1.75 g DHA: 1.10 g Duration: 8-wk	Yes, whole blood O3I: ↑ EPA: ↑ DHA: ↑	DXA FFM: ↔ FM: ↔	HGS ↑ CMJ Height ↔ Peak Power ↔ Mean Power ↔	Heterogeneous cohort No physical activity tracking
Gravina (93)	Male and female soccer players Total: *n*=26 PL: *n*=13 ω-3: *n*=13	Training log, included intensity and duration	Fish oil Total dose: 6.3 g∙d^−1^ EPA: 4.9 g DHA: 1.4 g Duration: 4-wk	Yes, whole blood ω-3: ↑	No measure	LEx_1RM_ ↔	Used weight-based dosing (0.1 g∙kg^−1^)
Hingley (51)	Male cyclists/runners Total: *n*=26 PL: *n*=13 ω-3: *n*=13	No intervention	Fish oil Total dose: 2.0 g∙d^−1^ EPA: 0.14 g DHA: 0.56 g Duration: 8-wk	Yes, RBC O3I: ↑ EPA: ↔ DHA: ↑	No measure	MVC: ↔ Power: ↔	
Crestani (86)	Adult men Total: *n*=15 PL: *n*=8 ω-3: *n*=7	PA log with duration and RPE, no prescription	Fish oil Total dose: 1.4 g∙d^−1^ EPA: 0.8 g DHA: 0.6 g Duration: 4-wk	Unmeasured	SKF FFM: ↔ FM: ↔	LEx_1RM_: ↔ LEx_Reps_: ↔	
Lewis (94)	Adult men Total: *n*=30 PL: *n*=12 ω-3: *n*=18	No intervention	Seal oil Total dose: 5 g∙d^−1^ EPA: 0.38 g DHA: 0.51 g Duration: 3-wk	Yes, plasma EPA: ↑ DHA: ↔	No measure	CMJ Height: ↔ SJ Height: ↔ Push-ups: ↔ BSQ_reps_: ↔	< 1 g∙d^−1^ LC ω-3 PUFA Short duration
Noreen (83)	Adult men and women Total: *n*=44 PL: *n*=22 ω-3: *n*=22	No intervention	Fish oil Total dose: 4 g∙d^−1^ EPA: 1.6 g DHA: 0.8 g Duration: 6-wk	Unmeasured	ADP FFM: ↑ FM: ↓	No measure	Only study to report increased LBM
Couet (84)	Adult men and women Total: *n*=6 (crossover trial)	No intervention	Fish oil Total dose: 6.0 g∙d^−1^ EPA: 1.1 g DHA: 0.7 g Duration: 3-wk	Unmeasured	DXA FFM ↔ FM ↓	No measure	Small sample Short duration Not randomized

BIA = Bioelectrical impedance analysis; BP_1RM_  = 1-repetition maximum bench press; BSQ_1RM_ = 1-repetition maximum back squat; DL_1RM_ = 1-repetition maximum deadlift; CMJ = counter movement jump; DXA = dual-energy X-ray absorptiometry; FFM = fat free mass; FM = fat mass; HTh_1RM_ = 1-repetition maximum hip thruster; LEx_1RM_ = 1-repetition maximum leg extension; LP_1RM_ = 1-repetition maximum leg press; MVC = maximum voluntary contraction; O3I = omega-3 index; RET = resistance exercise training; TT = time trial; RPP = rate pressure product; SJ = squat jump; SKF = skinfold calipers; TTF = time to fatigue; VAT = ventilatory anaerobic threshold; VO_2_max = maximal oxygen uptake; ↑ = significant increase; ↓ = significant decrease; ↔ = no significant difference.

#### 4.2.1. Body composition

One of the earliest studies employing RET with ω-3 PUFA supplementation randomized 28 healthy, untrained young females to one of three groups: RET only (*n* = 8), RET plus high-protein (30 g/day hydrolyzed whey protein) diet (HP) + 900 mg ω-3 PUFA (HP ω-3, *n* = 10), or RET plus HP + ω-3 PUFA + 5 grams/day creatine monohydrate (*n* = 10) [80]. The study lasted eight weeks, with four weeks devoted to pre-training and four weeks of RET plus the dietary intervention. Although the HP + ω-3 PUFA group experienced the greatest increase in FFM compared to RET only (1.35 kg vs 0.38 kg), this result was not statistically significant (*p* = 0.14). FM changes were similar between groups (RET: −0.39 kg, HP + ω-3: −0.35 kg). In a study of 18 healthy, trained young men, Georges et al. [81] found that eight weeks of periodized RET with concomitant intake of 3 g/d of krill oil (containing 393 mg EPA and 240 mg DHA) increased FFM (1.4 kg [2.1%]); however, this finding was not statistically significant compared to the control, despite a 1.1 kg difference. After eight weeks, no between or within-group differences were observed for FM (PL: 0.3 kg [0.3%], ω-3: −0.6 kg [−3.6%]). Similarly, a recent10-week RET trial in 21 men and women demonstrated that 4.5 g/d of fish oil (containing 2,280 mg EPA and 1.580 mg DHA) did not differentially increase FFM compared to PL (2.0 kg [3.4%] vs 1.4 kg [2.4%], *p* = 0.46). While FM changes were similar between groups (PL: 0.1 kg, ω-3: −1.0 kg, *p* = 0.09), it is notable that the between group difference was considered large (*d* = 0.84).

Studies employing aerobic training have reported no influence of ω-3 PUFA supplementation on FFM [52,82]. This finding is consistent with the minimal hypertrophic effects noted in aerobic studies. In Haghravan et al. [82], FFM was not preserved in either group (ω-3: −0.60%, PL: −0.34%). However, after eight weeks body fat percentage was significantly lower in the ω-3 group compared to PL (−1.24% vs − 0.33%, *p* = 0.009).

Of the ω-3 PUFA supplementation trials without a structured exercise regime, two reported significant body composition changes. Following 6 weeks of ω-3 PUFA supplementation (4 g/d, containing 1,600 mg EPA and 800 mg DHA), Noreen et al. [83] reported significant increases in FFM (0.5 kg vs −0.1 kg, *p* = .03) and decreases in FM (−0.5 kg vs 0.2 kg, *p* = .04) compared to PL. Couet et al. [84] observed a significant reduction in fat mass (−0.88 kg), and a non-significant increase in LBM (0.20 kg) following 3 weeks of an increased consumption of fish oil (6.0 g/d, containing 1,100 mg EPA and 700 mg DHA). No body composition changes were noted in two other trials [85,86].

Overall, only one study out of eight reported a statistically significant difference in FFM. While two additional studies reported results favoring ω-3 PUFAs by at least 1%, it is unclear if these changes were due to supplementation or other variables (e.g. training, diet). The evidence to date does not support a hypertrophic benefit for ω-3 PUFA supplementation with or without a structured resistance training program in young adults.

#### 4.2.2. Strength and power

In cross-sectional studies, ω-3 PUFA tissue status or dietary intake has been linked to improved upper- and lower-body strength, such as handgrip strength [87,88], knee extension strength [89], peak force production [90], and 1-repetition maximum (1RM) leg press [91]. In fact, EPA status has been correlated with strength and power measures in young adults [85,87]. Additionally, the positive relationship between ω-3 PUFAs and strength is a consistent finding in older adults [73,75,76]. In Hayward et al. [80], 1RM bench press, deadlift, squat, and hip-thruster increased across all groups after four weeks of supplementation. However, the HP + ω-3 PUFA (containing 540 mg EPA and 360 mg of DHA) group performed similarly to RET only. In another study [81], the ω-3 PUFA group experienced a modestly larger magnitude of increase in 1RM bench press (4.2 kg vs 3.4 kg) and leg press (49.0 kg vs 44.3 kg). In contrast, the most recent trial reported significantly higher absolute 1RM (11.3 kg vs 6.3 kg, *p* = 0.047) and relative 1RM bench press (*p* = 0.011) as well as higher relative 1RM back squat (*p* = 0.045) compared to PL. Of the trials without a structured training component, only one reported a significant difference in strength [92] while the others did not [51,86,93,94]. Interestingly, all trials failed to report differential effects on power [51,92,94].

Although plausible mechanisms exist for skeletal muscle hypertrophy following ω-3 PUFA supplementation, results in young adults are inconsistent and remain unconvincing. The primary metabolic driver of hypertrophy is increased MPS, specifically the myofibrillar proteins, in response to RET and protein feeding [95]. McGlory and colleagues [79] found that eight weeks of fish oil supplementation failed to influence the rates of MPS following 30 grams of whey protein ingestion with or without exercise in young trained men. Since this protein dose has been shown to maximize the rates of MPS [96], ω-3 PUFA supplementation is unable to enhance the effect beyond the already saturated muscle anabolic machinery. A recent cross-sectional analysis found that ω-3 PUFAs were associated with FFM, but only in those with low protein intake [97]. In agreement, Heileson et al. [98] observed that 10 weeks of RET plus 3,850 mg of EPA+DHA did not lead to significant differences in muscle hypertrophy compared to PL, in which participants reported an average daily protein intake ≥1.2 g∙kg^−1^. Indeed, this suggests that ω-3 PUFA supplementation may only enhance FFM when habitual dietary protein intake is suboptimal, in older individuals susceptible to aging anabolic resistance, or during periods of muscle disuse [31,99].

Data from previous trials suggests that ω-3 PUFA administration can increase strength; however, the process of ω-3 PUFA incorporation into the muscle cell may take a minimum of four weeks [36], then another three to six months until improvements in strength plateau [70,77]. While only two studies reported a significant group x time interaction for strength, trials >4 weeks are strongly associated with a treatment effect favoring ω-3 PUFA supplementation.

#### 4.2.3. Key findings for body composition, strength and power

- ω-3 PUFA supplementation may not confer a hypertrophic benefit in young adults.
- ω-3 PUFA supplementation may improve strength in a dose- and duration-dependent manner, although the effect may be attenuated with RET.
- More high-quality research is warranted to investigate the effects of ω-3 PUFA supplementation on body composition and physical performance outcomes.

### 4.3. Recovery and muscle soreness

A number of studies have assessed the effects of ω-3 PUFA supplementation with EPA and/or DHA on indices of skeletal muscle soreness (delayed onset muscle soreness [DOMS]), performance (strength and/or power output), range of motion (ROM), indirect measures of damage (creatine kinase [CK], lactate dehydrogenase [LDH]), and inflammatory markers (C-reactive protein [CRP], interleukin-6 [IL-6], tumor necrosis factor- α [TNF-α]) following exercise-induced muscle damage (EIMD) [100–112]. Collectively, results across studies suggest that DOMS may be reduced with ω-3 PUFA supplementation. For example, Heileson et al. [103] recently demonstrated that four grams of DHA or EPA consumed daily for seven weeks reduced subjective muscle soreness (as measured using a visual analogue scale; VAS) following 20 minutes of downhill running and jumping lunge exercises at 48-hour post-exercise in young healthy males compared to placebo. However, in the same study, a combined EPA/DHA supplement resulted in no statistically greater benefit compared to placebo in the measurement of DOMS. Another study demonstrated that subjective muscle soreness (as measured using VAS following a 60 minutes of downhill treadmill running) was decreased with four weeks of daily supplementation with an ω-3 PUFA supplement containing both EPA (2,145 mg) and DHA (858 mg) at 24-hours post-exercise when compared to placebo in young healthy males [105]. Furthermore, Lembke et al. [106] and Vandusseldorp et al. [111] showed participants reported DOMS was lower for up to 96 hours post-exercise in an ω-3 PUFA supplemented group versus placebo. Finally, a study done in rugby athletes supplementing with ω-3 PUFA demonstrated that lower body muscle soreness had a moderate beneficial effect during recovery when compared to a placebo supplement [100]. Although the studies discussed so far have found evidence of reduced muscle soreness following EIMD, there are also studies using ω-3 PUFA supplementation that have not found differences in this outcome which could be due to study design differences, dosing regimens, and the types of exercise used to induce EIMD [101,102,107,109,112].

While ω-3 PUFA supplementation has demonstrated some success in lowering subjective DOMS [100], evidence suggests that objective measures of performance in relation to skeletal muscle strength and power following EIMD are less robust. Rajabi et al. [108] showed that the daily ingestion of two grams of ω-3 PUFA for one month maintained leg press muscle strength in young healthy adults compared to a reduction for those receiving placebo. Furthermore, 7.5 weeks of ω-3 PUFA supplementation (6 g/d, containing 2.000 mg EPA and 1,800 mg DHA) reduced muscle damage 60 minutes after performing eccentric squat exercises, as measured by the maintenance of vertical jump performance which was similar to pre-supplementation levels [111]. Heileson et al. [103] also observed that the daily ingestion of four grams of DHA and EPA for seven weeks improved leg press muscle strength compared to placebo in young males. In contrast, others have shown no beneficial effects from ω-3 PUFA supplementation on measure of muscle strength [101,102,105–107,110,112]. Again, these inconsistent findings across studies may be related to methodological differences between study designs (i.e. dosing strategies, types of exercise used to promote EIMD). Further to this point, minimal evidence is available to suggest that joint of motion is significantly affected by ω-3 PUFA supplementation protocols [101,106,108,110].

Various indirect measures can be used to assess the degree of muscle damage through systemic blood-based biomarkers, such as CK and LDH. One study has confirmed that both CK and LDH were reduced 48- and 72-hours after EIMD in the ω-3 PUFA supplemented group when compared to placebo [108]; however, other studies have not demonstrated the same effect [101,102,105–107]. As ω-3 PUFA supplementation is known to have anti-inflammatory effects, certain studies have assessed systemic blood-based biomarkers of inflammation after muscle-damaging exercise in those supplementing ω-3 PUFA. While one study found a reduction in CRP 24 hours post-exercise [106], other studies were not able to demonstrate differences in IL-6, TNF-α, or CRP following EIMD in a variety of cohorts [101,103,105].

In summary, the evidence presented above indicates that ω-3 PUFA supplementation protocols are somewhat equivocal in whether they are able to reduce subjective DOMS following EIMD; however, other more objective markers of recovery following EIMD are shown to be less effective than hypothesized in a variety of study designs.

#### 4.3.1. Key findings for recovery

- ω-3 PUFA supplementation may attenuate indirect measures of muscle damage following intense exercise.
- ω-3 PUFA supplementation is equivocal in decreasing subjective measures of muscle soreness following intense exercise.
- ω-3 PUFA supplementation does not decrease measures of inflammation following exercise-induced muscle damage.

### 4.4. Immune health

While moderate exercise improves immune health, athletes who undergo high volumes of intense training are at a higher risk of developing illnesses such as upper respiratory tract infections (URTI). For instance, a 2024 study by Post et al. [113] reported that respiratory illness was the most common type of illness reported by Team USA athletes during the 2023 Pan American games. Similarly, Soligard et al. [114] reported that of the 651 illnesses reported during the 2016 Olympic Games, 47% impacted the respiratory system while 21% impacted the gastrointestinal system. Moreover, endurance athletes such as ultramarathon runners and long-distance triathletes are frequently impacted by challenges to their immune system [115,116]. URTI can disturb training programs and will inevitably hinder performance in training and/or competition. Therefore, strategies to mitigate the immunological stress induced by high-volume training should be implemented. Multiple nutritional ingredients have been researched for their ability to support and enhance the resilience of the immune system [117,118]. In this regard, ω-3 PUFA influence both innate and adaptive immune cells. ω-3 PUFAs have the ability to regulate cell signaling processes and are an integral part of the cellular membrane that can provide membrane fluidity while also impacting the assembly of lipid raft complexes [119,120], microstructures within cells with a particular rich distribution of lipids. ω-3 PUFA-derived metabolites such as prostaglandins, leukotrienes, thromboxanes, maresins, protectins, and resolvins are integral immune-regulatory molecules known as specialized pro-resolving mediators (SPMs) that can affect the inflammatory response to an immune stressor [119]. Multiple reviews cover the beneficial effects of ω-3 PUFA on the immune system and how they affect immune-related diseases such as chronic inflammatory disease, type I diabetes, and responses to bacterial and viral infections [121–124]. In general, ω-3 PUFA decrease macrophage cytokine expression, increase macrophage and neutrophil phagocytosis to enhance microbial clearance, decrease activation of basophils, mast cells, and T cells, and have other effects on the various immune cell types [119]. Additionally, ω-3 PUFAs from fish oil have the ability to upregulate immune cell function by stimulating CD4 and CD8 lymphocytes, which help ward off pathogens [125]. Among other benefits, fish oil can provide beneficial immunomodulatory effects for infants when consumed postnatally [126,127], provide benefit for individuals who suffer from arthritis who cease nonsteroidal anti-inflammatory drug (NSAID) usage [128], improve innate immunity [129], promote anti inflammation [130], and can augment post-exercise immune function [131].

When considered in the context of challenging exercise or as part of an ongoing exercise stimulus, several studies have suggested that ω-3 PUFA availability enhances immune support. As one considers exercise-induced stress on the immune system, ω-3 PUFA acids reduce inflammation and oxidative stress following exercise bouts [132]. Along these lines, de Lourdes Nahas Rodacki et al. [77] reported that supplementing elderly women who performed strength training with two grams per day of fish oil for 150 days experienced similar immune cell responses in addition to also decreasing TNF-α, interferon-gamma (IFN-γ), IL-2, and IL-6, while IL-10 increased. In athletes, most of the immune outcomes assessed have been in the production of cytokines from immune cells. Table 3 shows evidence from studies with ω-3 PUFA supplementation’s effects on athlete immune response [133–144].

**Table 3.** ω-3 PUFA effects on Athlete’s immune health.

Author	Athletes	Protocol/Season	Diet Control	Duration/Dose	Timing	Outcome
Andrade et al. [133]	Elite male swimmers	Pre-season	Yes	6 wks/950mgEPA, 500mgDHA	Daily	↑ plasma LCFAs; ↓ plasma AA, PGE2; ↔ TNF-ɑ, IL-2, IL-4;
Delfan et al. [138]	Elite male paddlers	4-wk. sculling training	Yes	4 wks/2.4gEPA, 1.2gDHA	Daily	↓ TNF-ɑ, IL-1β, IFN-*γ*; ↔ IL-4; ↑ IL-10; Th to Th2
Santos et al. [144]	Competitive male marathon	60-Days prior to marathon	No	60d/300mgEPA, 1500mg DHA	Daily, pre-marathon	↑ pre-and post-race lymphocyte proliferation; ↓ TNF-ɑ, IL-2, IL-10 pre-race; ↔ TNF-ɑ, IL-2, IL-10 post-race
Nieman et al. [142]	Competitive male and female cyclists	3 Days of 3 hrs cycling at 57% W_max_	Yes	6 wks/2000mg EPA,400mgDHA	Twice daily	↑ plasma EPA, DHA; ↔ TT, plasma cytokines, MP, blood leukocytes, CRP, CK
Da Boit et al. [145]	Competitive male and female cyclists	Training	No	16 wks/550 mg EPA, 550 mg DHA	Twice daily	↔ URTI incidence, severity, duration, sIgA; ↓ symptom days
Capó et al. [134]	Semipro male soccer	In-season competition and training	No	8 wks/1.14gDHA	Daily, prior to exercise	↓post-ex MIP1-α, TNF-α, IL-6, TLR-4, GP, ROS; ↑PBMCs, UCP-3, SOD
Capó et al. (135)	Semipro male soccer	In-season competition and training	No	8 wks/1.14gDHA	Daily, prior to exercise	↑ blood DHA, ↑PMN gene expression, CAT activity, total MP; ↔ PMN: NO_3_^−^, NO_2_^−^; NO; MP, COX_2_, TNF-α, gene expression
Price et al. (143)	Recreational male and female endurance w/EIB	Pre-and post-spirometry tests	No	3 wks/3000mg EPA, 3000mg DHA	Daily	↔ post-EVH ΔFEV_1max_
Mickleborough et al. [141]	Elite male and female endurance	Pre- and post-ex. PFT, immune analysis	Yes	3 wks/3200mg EPA, 2200mg DHA	Daily	↓ pre- and post-ex. LTE_4_, 9ɑ, 11β PGF_2_, LTB_4_, TNF-α, and IL-1β
Marques et al. [140]	Male wheelchair basketball	30d, 4x/wk, 4hr training sessions	Yes	30 d/300mg EPA, 1500mg DHA	Daily	blunted post-ex. ↑ LDH, IL-6; maintained PMN integrity
Macartney et al. [139]	Three elite cyclists	During sea son and Tour de France	Yes	12 wks/559-1118mg EPA, 229-458mg DHA		↑ O3I pre-, during Tour de France

CAT = catalase; COX2 = Cyclooxygenase-2; CK = creatine kinase; CRP = C-reactive protein; EVH = eucapnic voluntary hyperpnea; FEV1 = forced expiratory volume in 1 second; GP = glutathione peroxidase; IFN-*γ* = interferon-gamma; IL = interleukin; LMH = lactate dehydrogenase; LTB = leukotriene B4; LTE = leukotriene E4; PBMCs = peripheral blood mononuclear cells; PEG2 = prostaglandin E2; PMN = polymorphonuclear cells; MP = myeloperoxidase; slgA = secretory immunoglobulin A; MIP1-α = macrophage inflammatory protein-1 alpha; NO = nitric oxide; O3I = omega-3 index; ROS = reactive oxygen species; SOD = superoxide dismutase; Th = T helper cells; TLR-4 = toll-like receptor 4; TNF-ɑ = tumor necrosis factor-alpha; TT = time trial; UCP-3 = uncoupling protein 3; URTI = upper respiratory tract infection; ↑ = significant increase; ↓ = significant decrease; ↔ = no significant difference.

A variety of immune-modulating cytokines from various immune cells are affected; however, due to methodological differences it is difficult to formulate substantial conclusions on fish oil effects on immune response. A recurrent finding is the reduction in TNF-α after at least four weeks of 2,400 mg EPA and 1,200 mg DHA from fish oil supplementation, which could also be seen with higher dosages for a shorter period of time or lower dosages for eight weeks [133,138,141,144]. Additionally, post-exercise/competition pro-inflammatory immune response is attenuated after chronic fish oil supplementation [134,136,141,144], which could be beneficial for athletes competing in multistage/event sports. In addition, krill oil supplementation has also shown to enhance peripheral blood mononuclear cell (PBMC)-derived interleukin-2 and natural killer cytotoxic activity after a cycling time trial when krill oil was supplemented for six weeks [145]. Though cod liver oil can improve clinical outcomes for children suffering from URTI [146], in the current state of the evidence, fish oil supplementation does not seem to produce any beneficial effects on URTI incidence in athletes due to the paucity of research. Da Boit et al. [137] found no differences in URTI incidence, duration, and severity after 16 weeks of ω-3 PUFA supplementation in trained cyclists; however, this study also incorporated vitamin D and whey protein in the experimental group. To our knowledge, no other studies have examined ω-3 FA consumption and URTI incidence.

Exercise-induced bronchoconstriction (EIB), formerly referred to as exercise-induced asthma, is another malady experienced by some highly trained athletes. EIB is characterized by coughing, wheezing, and breathlessness due to bronchoconstriction, which can be induced by hyperpnea in environments with cold, dry air [147]. Endurance athletes who typically undergo high-volume training are more susceptible to EIB due to the high stress placed on the immune system and the rapid ventilatory responses to exercise. Epidemiological research suggests possible benefits of ω-3 PUFAs for asthma mitigation in infancy and/or childhood due to their anti-inflammatory properties [148–152]. However, effects of ω-3 PUFA consumption through fish or fish oil on asthma symptoms in adults are inconsistent [153–156]. It is theorized that ω-3 PUFA consumption can ameliorate or mitigate symptoms of asthma in sporting conditions; however, results in athletes are scarce. In a randomized, crossover manner, Mickleborough [141] assessed if 3,200 mg EPA and 2,200 g DHA in fish oil could mitigate EIB onset in elite male and female endurance athletes and found improved postexercise pulmonary function in the ω-3 PUFA group compared to the control group. One study in healthy males and females prone to hyperpnea-induced bronchoconstriction (HIB) showed similar reductions in HIB symptoms as montelukast, a pharmacotherapy for asthma [157]. Contrarily, a pilot study showed no effects on post-eucapnic voluntary hyperpnea in recreational athletes who supplemented with fish oil for six weeks [143]. Further research must be conducted on ω-3 PUFA consumption in athletes to assess immunomodulation before or after exercise. Consistency in methodologies would simplify interpretations of results in future studies.

#### 4.4.1. Key findings for immune health

- Many athletes can develop a compromised immune system due to the stress of high training volumes, which can increase the likelihood of developing acute respiratory infections that negatively impact their ability to train and compete.
- ω-3 PUFA supplementation can affect various immune cell responses in non-athlete, clinical, and athletic populations.
- Many studies conducted in athletic populations have indicated that ω-3 PUFA supplementation can influence the production and regulation of various inflammatory cytokines, which may lead to further physiological consequences for the athlete.

### 4.5. Cognitive and psychological health

More than half of the brain’s composition is made up of lipids and approximately one-third of those lipids are ω-3 PUFAs, with DHA and AA being the predominant fatty acids. These fatty acids are directly linked to the development of the central nervous system (CNS) and neural function in neonates, as they are transferred through the placenta [158]. Due to the limited conversion of dietary ALA to DHA, supplementation with DHA is essential during pregnancy and even after birth [159]. Deficiencies in ω-3 PUFAs can hinder neonatal and infant growth and development, potentially leading to neurological diseases, memory impairment, and difficulties in learning and processing [160]. Brain function and cell growth extend beyond prenatal development and are crucial during the first few years of an infant’s life. After pregnancy, it remains essential for infants to ingest ω-3 PUFAs for brain development, either through mother’s milk or formula enriched with DHA and AA [161,162]. Breastfeeding mothers who consumed 200 mg of DHA per day for four months gave birth to infants with higher psychomotor functioning and improved hand-eye coordination at 30 months of age [163]. In contrast, inadequate intake of ω-3 PUFAs can lead to stunted learning and cognitive deficits [164].

ω-3 PUFAs play an essential role in the phospholipid bilayer of the cell membrane, affecting membrane fluidity and function, which are vital for cellular transport and communication [165–167]. ω-3 PUFAs can also influence neurotransmitter regulation. Diets low in ω-3 PUFAs have been associated with reduced levels of serotonin and dopamine [168]. It is believed that ω-3 PUFAs contribute to the composition of membranes, enhancing organization, elasticity, and permeability, which can facilitate neurotransmitter and glucose uptake in the brain [169]. EPA has been hypothesized to possess neuroprotective qualities due to its antioxidant and anti-inflammatory properties. It has been reported that larger doses of EPA and DHA dietary intake reduce platelet aggregation [170] and blood pressure [171]; it stands to reason that ω-3 PUFAs may impact cerebral blood circulation, given their ability to cross the blood-brain barrier. It is commonly recognized that increasing cerebral blood flow can increase the delivery of oxygen and nutrients to the brain, which can affect cognitive function and mental health. One study conducted an oxygenation and mood measurement in healthy females during an arithmetic test with ω-3 PUFAs supplementation and found that EPA was positively associated with increases in cerebral blood flow and inversely correlated with negative moods related to depression and dejection [172]. This suggests that ω-3 PUFAs, specifically EPA, may assist in increasing oxygenation levels in the brain while also coinciding with increasing parameters of psychological performance. Several of these mechanisms could explain why ω-3 PUFAs could potentially enhance cognitive function relevant to athletic performance, including attention, memory, reaction time, and decision-making. Additionally, ω-3 PUFAs may support recovery from intense exercise by reducing inflammation, which could improve sleep and indirectly benefit cognitive function. Additionally, ω-3 PUFAs have been shown to improve stress resistance, reduce anxiety, and enhance mood.

Most studies on the effects of ω-3 PUFAs on cognitive function have focused on children, individuals with dementia, Alzheimer’s disease (AD), mild cognitive impairment (MCI), age-related cognitive decline, and elderly populations. Alternatively, a limited number of studies have investigated these outcomes in healthy young athletes (Table 4).

**Table 4.** ω-3 PUFAs studies on cognition and psychological health in athletes.

Author	Athletes	Protocol/Season	Diet Control	Duration/Dose	Timing	Outcome
Fontani et al. [173]	Male and female athletes	Cognitive function tests, alertness; GNG, SAT ReT; Choice, EEG, EMG	No	5 wks/1600 mg EPA, 800 mg DHA	Daily	↑ vigor, ↓ anger, anxiety, depression; ↓ ReT in GNG, SAT; EEG theta, alpha; ↓ HC; ↓ AA/EPA ratio
Fontani et al. (2009)	Male and female karate	POMS, alertness, GNG, SAT ReT, EEG, EMG	No	3 wks/1200mg EPA, 600 mg DHA	Daily	↓ ReT, latency of MRBMPs; ↑ vigor
Guzmán et al. (2011)	Elite female soccer	in-season competition and training	Yes	4 wks/3500 mg DHA	Daily	↓ complex ReT; ↑ efficiency
Black et al. [100]	Professional male rugby Union	Preseason training RT, FBT	Yes	5 wks/551 mg EPA, 551 mg DHA	Twice daily	↑ sleep quality, ↓ fatigue

EEG = electroencephalography; EMG = electromyography; FBT = field-based training; GNG = go-no-go; HC = homocysteine; MRBMPs = movement-related brain macropotentials; ReT = reaction time; SAT = sustained attention test.

Fontani and colleagues [173] assessed the effects of ω-3 PUFAs on cognition in healthy adults using a computerized cognitive battery of tests, along with physiological responses measured through electroencephalogram (EEG) and electromyography (EMG) readings. Following 35 days of supplementation, blood levels showed a reduction in AA:EPA ratio with improvements in overall mood states of anger, anxiety, and depression. Cognitive function tests revealed a decrease in reaction time and a shift toward theta and alpha waves from the EEG. This is important for distinguishing mental functioning in healthy individuals by examining both cognitive and physiological responses to ω-3 PUFA supplementation. However, this study had limitations that may have implications for the findings, such as small and inconsistent sample size and non-counterbalanced groups.

A double-blind, counterbalanced crossover study examined the effects of EPA or DHA supplementation on cognition in young, healthy adults using functional magnetic resonance imaging (fMRI). Participants completed Stroop and working memory tasks both before and after 30 days of supplementation. While both groups reduced AA:EPA ratio levels, the EPA group showed a reduction in the anterior cingulate cortex and increases in precentral gyrus activation during reductions of the reaction time during the Stroop test, while DHA supplementation increased right precentral gyrus activation during Stroop and working memory tests. The results indicated that, although there were differences in brain activation and cognitive performance between the respective supplements, both showed cognitive changes following 30 days of supplementation [174].

ω-3 PUFAs have also been evaluated in specific domains of cognitive functioning and executive functioning related to impulsivity. The results of cognitive testing and mood assessments indicated few effects, with ω-3 PUFAs associated with a decrease in risk-averse decisions. However, the findings suggest that ω-3 PUFAs may influence decision-making without being directly linked to impulsiveness [175]. In randomized controlled trials with larger sample sizes measuring cognition and executive function with ω-3 PUFA supplementation, results after 18 weeks were inconclusive regarding improvements in cognitive domains. However, some participants did show improved executive function, particularly those with initially low DHA levels [176]. Age does not appear to influence the potential benefits of ω-3 PUFA supplementation on cognitive performance outcomes, particularly in memory and executive function, throughout adulthood [177]. This highlights the inconsistency and uncertainty in assessing the effects of ω-3 PUFAs on cognition within a healthy, young demographic.

Limited studies are available measuring the efficacy of ω-3 PUFA on cognitive function and mood in non-diseased or clinically diagnosed populations. While the mechanisms underlying changes in cognitive function and mood have been studied frequently in both humans and animals, further research is needed on individuals who are healthy, young, and free from any neurological or mental diagnoses. This research is essential to better understand the potential benefit ω-3 PUFA supplementation has on mental functioning and mood in an athletic population.

#### 4.5.1. Key findings for cognitive and psychological health

- ω-3 PUFAs are crucial for optimal brain development and functioning.
- ω-3 PUFA supplementation can increase membrane fluidity, neurotransmitter synthesis and release, and cerebral blood flow.
- Studies in healthy, young athletes assessing the different theorized improvements in sport-specific cognitive functions are needed.

### 4.6. Traumatic brain injury

Globally, an estimated 69 million individuals experience a traumatic brain injury (TBI) each year [178]. A concussion is a type of TBI that is defined as a direct or indirect impact to the head causing neurometabolic dysregulation that is followed by a range of symptoms which occur without the presence of a skull fracture and with negative findings on conventional neuroimaging (e.g. magnetic resonance imaging [MRI]) [179,180]. It is the cascade of neurometabolic events that are purported to cause cognitive dysfunction and physical symptoms [181]. Despite the high prevalence of concussions, limited effective treatment options are currently available and presently there are no dietary strategies or supplements that have been approved to aid with concussion recovery [182].

The body of evidence is growing that demonstrates nutritional strategies, such as ω-3 PUFA supplementation, can alter the neurometabolic cascade following TBI [183–185]. DHA is found in high concentrations in neuronal cells and contributes to maintaining brain function [186] and membrane integrity [187], while EPA acts in an anti-inflammatory manner [188].

Multiple pre-clinical animal models have supported the benefits of a diet enriched with ω-3 PUFAs to improve TBI related cognitive and neurophysiological outcomes [189–196]. ω-3 PUFAs provided through the diet or through injections reduced or attenuated neuroinflammation, neuronal death, cerebral edema, and behavioral deficits compared to placebo [189–192,194]. In addition, increased ω-3 PUFA concentrations in the brain are positively associated with time to first movement and improved neurological severity scores 24-hours post-TBI [197]. Mechanistically, ω-3 PUFA are purported to function as an antioxidant, thereby attenuating ROS induced by TBI [193,198], upregulating brain derived neurotrophic factor (BDNF) [196,199], and decreasing stress resistance and synaptic dysfunction, which may influence cognitive function [195,196]. In support of these mechanisms, ω-3 supplementation administered before or after a TBI can improve cognitive performance compared to controls in rodent models [192,194,199–201] with higher DHA doses revealing greater benefits [201]. These cognitive benefits can be observed early after TBI where Morris water maze performance and beam walking scores are improved as early as day 1 and day 2 post-TBI, respectively [185]. Overall, multiple purported pathways and positive cognitive findings in animal models have demonstrated that ω-3 PUFA supplementation could improve recovery following TBI. Despite the promising pre-clinical findings, limited human clinical trials have evaluated the efficacy of ω-3 PUFA supplementation on concussions [184,185].

Currently, three studies have investigated the prophylactic use of ω-3 PUFA supplements in American football players (a sport characterized to involve repeated head impacts and has a higher prevalence of concussions) [202–207]. Oliver et al. [204] performed a randomized controlled trial where Division I NCAA American football players (*N* = 81) received either placebo, 2,000 mg/d DHA, 4,000 mg/d DHA, or 6,000 mg/d DHA beginning prior to the off-season training until the conclusion of the competitive season (total of 189 days). The DHA supplement increased the proportion of plasma DHA in the fatty acid profile in a dose dependent manner. When results were collapsed across all treatment conditions, DHA attenuated increases in serum neurofilament light (Nf-L; a marker of axonal damage) compared to placebo. Another multi-site non-randomized trial had a Division I team supplement with ω-3 PUFAs (containing 2,000 mg/d DHA, 560 mg/d EPA, and 320 mg/d DPA) and a Division III team act as a control [202]. Nf-L increased over the season in the control team, while there was no change over time in those receiving the ω-3 PUFA supplement. These findings suggest that prophylactic ω-3 PUFA supplementation may be neuroprotective for repeated head impacts. In contrast, Mullins et al. [203] found that ω-3 PUFA supplementation (2442 mg/day DHA, 1020 mg/day EPA five days per week for 26 weeks), compared to placebo, did not attenuate the Nf-L increases or influence inflammatory cytokines across a NCAA Division I football season (*n* = 38). A recent meta-analysis of the three previously discussed studies found that, taken together, their results show ω-3 PUFA supplementation results in lower Nf-L concentrations at the end of the college football season compared to placebo (mean difference = −2.23 ± 0.83 pg⋅mL^−1^) [208]. Table 5 outlines studies that have assessed changes in traumatic brain injury with omega-3 polyunsaturated fatty acid supplementation

**Table 5.** The effect of prophylactic ω-3 PUFA supplementation on traumatic brain injury in American football players.

Author	Athletes	Protocol/Season	Diet Control	Duration/Dose	Timing	Outcome
Oliver et al. [204]	NCAA DI football	In-season competition and training	Yes	27 wks/2g DHA, 4g DHA or 6g DHA	Daily	↑ plasma DHA; ↓ Nf-L
Heileson et al. [202]	NCAA DI football	In-season competition and training	Yes	131 days/2000mg DHA, 560mg EPA, 320mg DPA	4 times per week	↑ plasma DHA; ↑ plasma EPA; ↓ Nf-L
Mullins et al. 203	NCAA DI football	Pre-, in-and post-season competition and training	No	26 wks/EPA:1,000g DHA:2,400g	Daily	↑ plasma DHA; ↑ plasma EPA; ↔ Nf-L

Nf-L = neurofilament light.

Currently, one randomized controlled trial is available that has investigated DHA supplementation (2 g/day) following a sport-related concussion in adolescents (*n* = 40; 14–18 years of age). No statistically significant difference was identified between groups for recovery times was found [209]. However, DHA supplementation resulted in participants being symptom-free five days earlier, and participants were able to begin the return to sport progression 4.5 days sooner than controls, which may be clinically meaningful. Future larger randomized controlled trials are urgently warranted to determine the efficacy of ω-3 PUFA supplementation.

#### 4.6.1. Key findings for traumatic brain injury

- Approximately 69 million individuals experience TBIs globally each year.
- ω-3 PUFA supplementation may positively influence the neurometabolic cascade following TBIs, reducing neuroinflammation and cognitive dysfunction in animal models.
- In humans, a limited amount of evidence suggests that prophylactic ω-3 PUFA supplementation may offer neuroprotective benefits in athletes following repeated head impacts.

### 4.7. Sleep

Sleep is one of the crucial factors influencing the performance of athletes. Sufficient sleep is critical for muscle repair and recovery, and adequate sleep has been linked to improved athletic performance. Sleep is also important as it supports the immune system function, and helps to regulate mood and motivation to exercise. Deficiencies of ω-3 PUFAs in the diet have been linked to disturbances in circadian rhythm, sleep disturbances, and melatonin rhythm [210]. ω-3 PUFA levels can influence melatonin synthesis, where low levels of ω-3 PUFA consumption can decrease melatonin secretion [211]. The anti-inflammatory properties of ω-3 PUFAs reduce the risk of chronic diseases [212] and have also shown improvements in memory impairment in sleep-deprivation in rats [213]. DHA has been shown to directly affect sleep regulation, specifically in situations where there is a insufficiency in ω-3 PUFAs. A reduction in ω-3 PUFA intake through the diet results in a 30–50% decrease in DHA membrane content [214] and inconsistent sleep patterns [210]. Fatty fish consumption, a primary dietary source of ω-3 PUFAs, has been associated with sleep latency, daily functioning, heart rate variability (HRV) [215], and better quality of sleep [216]. Restricted ω-3 PUFA consumption, and the resulting decreased DHA concentration, can reduce the metabolism of dopamine and serotonin [212]. This could explain why ω-3 PUFAs are beneficial in treating major depressive disorders and result in decreased symptoms of depression, anxiety, and improved emotional regulation [217]. ω-3 PUFA contribution to combatting depression symptoms, including sleep disturbances, can promote healthy sleep cycles. Dashti et al. [218] concluded that longer sleep duration correlates with lower BMI and reduced saturated fat intake in young adults, which supports the findings of shorter sleep cycles correlating with low levels of diet quality, specifically in EPA and DHA [219]. Still, limited research has been completed on the effects of EPA alone on sleep. However, EPA affects the production of prostaglandins, prostaglandin D2 specifically, which mediates sleep and wake cycles [220]. Future research is needed on EPA’s effects on sleep regulation.

There has been some association between ω-3 PUFA levels and Obstructive Sleep Apnea Syndrome (OSA). Low levels of DHA and EPA have been associated with OSA [221], which often results in elevated inflammatory markers [222] and cardiovascular comorbidities [223]. Concentrations of ω-3 PUFAs in patients with obesity who suffer from OSA showed a positive relationship between sleep efficiency and rapid eye movement (REM) sleep [224]. However, in patients with chronic insomnia ω-3 PUFA supplementation had no effect on the quality of sleep, sleep-wake cycle, or melatonin production [225]. The effects of ω-3 PUFA supplementation on sleep outcomes in individuals with clinical sleep disorders has not been fully elucidated and more research is required.

Some positive effects have been observed with dietary ω-3 PUFA supplementation in healthy populations with no preexisting sleep disorders or comorbidities [226]. Sixty days of 2.5 g/d of EPA plus DHA supplementation is sufficient to decrease daytime sleepiness in deployed U.S. soldiers [227]. Children’s sleep research findings show that increases in ω-3 PUFA DHA can reduce wake times during the night and improve overall sleep wellness [228]. Similar findings have been observed in healthy adolescents where improved sleep timing and sleep duration have been observed with ω-3 PUFA supplementation, specifically when supplementation induces increased plasma DHA levels [229].

#### 4.7.1. Key findings for sleep

- ω-3 PUFA supplementation has been linked to improved sleep quality in some studies.
- ω-3 PUFA supplementation may help increase sleep quality due to anti-inflammatory properties and effects on neurotransmitters like dopamine and serotonin, a precursor of melatonin, which helps regulate sleep-wake cycles.
- However, inconsistency of results indicates more research is needed to fully understand the relationship between ω-3 PUFA supplementation and sleep.

### 4.8. Gut health

The classic definition of a prebiotic refers to non-digestible carbohydrates that specifically support the growth of health-promoting bacteria that colonize the host’s gastrointestinal tract [230]. However, based on recent scientific advances and clinical research, the International Scientific Association for Probiotics and Prebiotics (ISAPP) updated its definition of a prebiotic in 2017 to potentially include non-carbohydrate substances, such as ω-3 PUFAs [231]. A prebiotic is now defined as a substrate that is selectively utilized by host microorganisms, conferring a health benefit [231]. For a substance to be considered a prebiotic, it must be utilized by live microorganisms in a way that improves host health.

**Figure 2.**
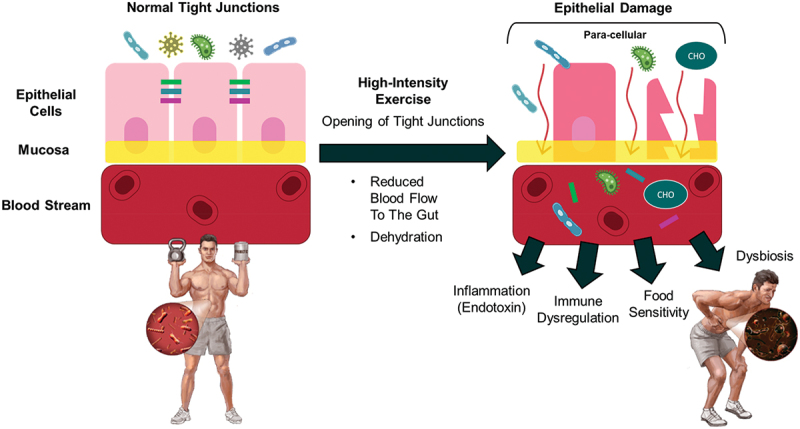
Prolonged maximal exercise can induce leaky gut (adapted from Dr. Jeremy Townsend).

Exercise-induced gastrointestinal symptoms are common across many sports, particularly in endurance events. In long-distance runners, cyclists, and triathletes, the prevalence of these symptoms can reach up to 70% [232]. Commonly reported symptoms include diarrhea, vomiting, nausea, and abdominal cramping [233]. During maximal exercise, splanchnic blood flow can be reduced by as much as 80% [234], as blood is redirected from the gut to the exercising muscles to meet the increased demand for oxygen and nutrients. This shift in blood flow can lead to the opening of tight junctions in the gut lining, which increases mucosal permeability and may allow harmful substances to enter the bloodstream (Figure 2). Consequently, this can trigger increased inflammation and contribute to gut dysbiosis [235].

Compared to sedentary individuals, athletes tend to have a gut microbiota with a higher abundance of health-promoting bacterial species, increased microbial diversity, and enhanced functional metabolic capacity. Exercise also stimulates the growth of bacteria that can modulate mucosal immunity and improve gastrointestinal barrier function [236]. Additionally, supplementation with probiotics (live bacteria) has been shown to support immune and digestive health in athletes [237].

ω-3 PUFA intake and circulating levels have been linked to improvements in gut microbiome composition, particularly an increase in alpha diversity, which refers to the variety of microbial species in the gut [238,239]. ω-3 PUFAs also promote increases in the abundance of *Lachnospiraceae*, a family of bacteria that are among the most abundant taxa in the gut microbiota [239]. *Lachnospiraceae* are known for their anti-inflammatory properties and play a key role in maintaining the integrity of the gut barrier through the production of short-chain fatty acids such as butyrate and acetate [239,240]. Butyrate plays a critical role in gut health by serving as an energy source for colonocytes. It also exerts anti-inflammatory and immune-modulating effects and helps maintain the intestinal barrier function. Butyrate supports gut epithelial cell proliferation and differentiation, further promoting a healthy gut environment and potentially providing protection from exercise-induced leaky gut.

#### 4.8.1. Key findings for gut health

- ω-3 PUFA are prebiotics and supplementation may improve the composition of the gut microbiome
- High-intensity exercise might cause leaky gut resulting in inflammation and gut dysbiosis.
- While early studies indicate potential benefits of ω-3 PUFA supplementation on gut microbiome composition, studies in exercising athletes are needed.

## 5. Final summary and conclusions

The following 10 points constitute the Position Statement of the Society. They have been approved by the Research Committee of the Society:

1. Athletes may be at a higher risk for ω-3 PUFA insufficiency.
2. Diets rich in ω-3 PUFA, including supplements, are effective strategies for increasing ω-3 PUFA levels.
3. ω-3 PUFA supplementation, particularly eicosapentaenoic acid (EPA) and docosahexaenoic acid (DHA), has been shown to enhance endurance capacity and cardiovascular function during aerobic-type exercise.
4. ω-3 PUFA supplementation may not confer a muscle hypertrophic benefit in young adults.
5. ω-3 PUFA supplementation in combination with resistance training may improve strength in a dose- and duration-dependent manner.
6. ω-3 PUFA supplementation may decrease subjective measures of muscle soreness following intense exercise.
7. ω-3 PUFA supplementation can positively affect various immune cell responses in athletic populations.
8. Prophylactic ω-3 PUFA supplementation may offer neuroprotective benefits in athletes exposed to repeated head impacts.
9. ω-3 PUFA supplementation is associated with improved sleep quality.
10. ω-3 PUFA are classified as prebiotics; however, studies on the gut microbiome and gut health in athletes are currently lacking.
